# Structural and functional hepatocyte polarity and liver disease

**DOI:** 10.1016/j.jhep.2015.06.015

**Published:** 2015-10

**Authors:** Paul Gissen, Irwin M. Arias

**Affiliations:** 1 MRC Laboratory for Molecular Cell Biology, University College London, London, UK; 2 UCL Institute of Child Health, London, UK; 3 Great Ormond Street Hospital, London, UK; 4 Cell Biology and Metabolism Program, Eunice Kennedy Shriver National Institute of Child Health and Human Development, National Institutes of Health, United States

**Keywords:** TJ, Tight junctions, BC, Bile canaliculi, ATP, Adenosine triphosphate, ABC, ATP binding cassette, TGN, Trans Golgi Network, RE, Recycling endosomes, ECM, Extracellular Matrix, cAMP, Cyclic Adenosine Monophosphate, PKA, Protein Kinase A, GPI, Glycophosphatityl Inositol, SNARE, Soluble N-ethylmaleimide-sensitive factor attachment Protein Receptor, ARC, Arthrogryposis Renal dysfunction and cholestasis syndrome, MVID, Microvillus Inclusion Disease, NISCH, Neonatal ichthyosis-sclerosing cholangitis syndrome, FHC, Familial hypercholanemia, HBV, Hepatitis B Virus, HCV, Hepatitis C virus, Hepatocyte polarity, Inherited liver diseases, Hepatocyte biology, Cholestasis, Canalicular diseases

## Abstract

Hepatocytes form a crucially important cell layer that separates sinusoidal blood from the canalicular bile. They have a uniquely organized polarity with a basal membrane facing liver sinusoidal endothelial cells, while one or more apical poles can contribute to several bile canaliculi jointly with the directly opposing hepatocytes. Establishment and maintenance of hepatocyte polarity is essential for many functions of hepatocytes and requires carefully orchestrated cooperation between cell adhesion molecules, cell junctions, cytoskeleton, extracellular matrix and intracellular trafficking machinery. The process of hepatocyte polarization requires energy and, if abnormal, may result in severe liver disease.

A number of inherited disorders affecting tight junction and intracellular trafficking proteins have been described and demonstrate clinical and pathophysiological features overlapping those of the genetic cholestatic liver diseases caused by defects in canalicular ABC transporters. Thus both structural and functional components contribute to the final hepatocyte polarity phenotype. Many acquired liver diseases target factors that determine hepatocyte polarity, such as junctional proteins. Hepatocyte depolarization frequently occurs but is rarely recognized because hematoxylin-eosin staining does not identify the bile canaliculus. However, the molecular mechanisms underlying these defects are not well understood. Here we aim to provide an update on the key factors determining hepatocyte polarity and how it is affected in inherited and acquired diseases.

## Introduction

**Fig. 1:**
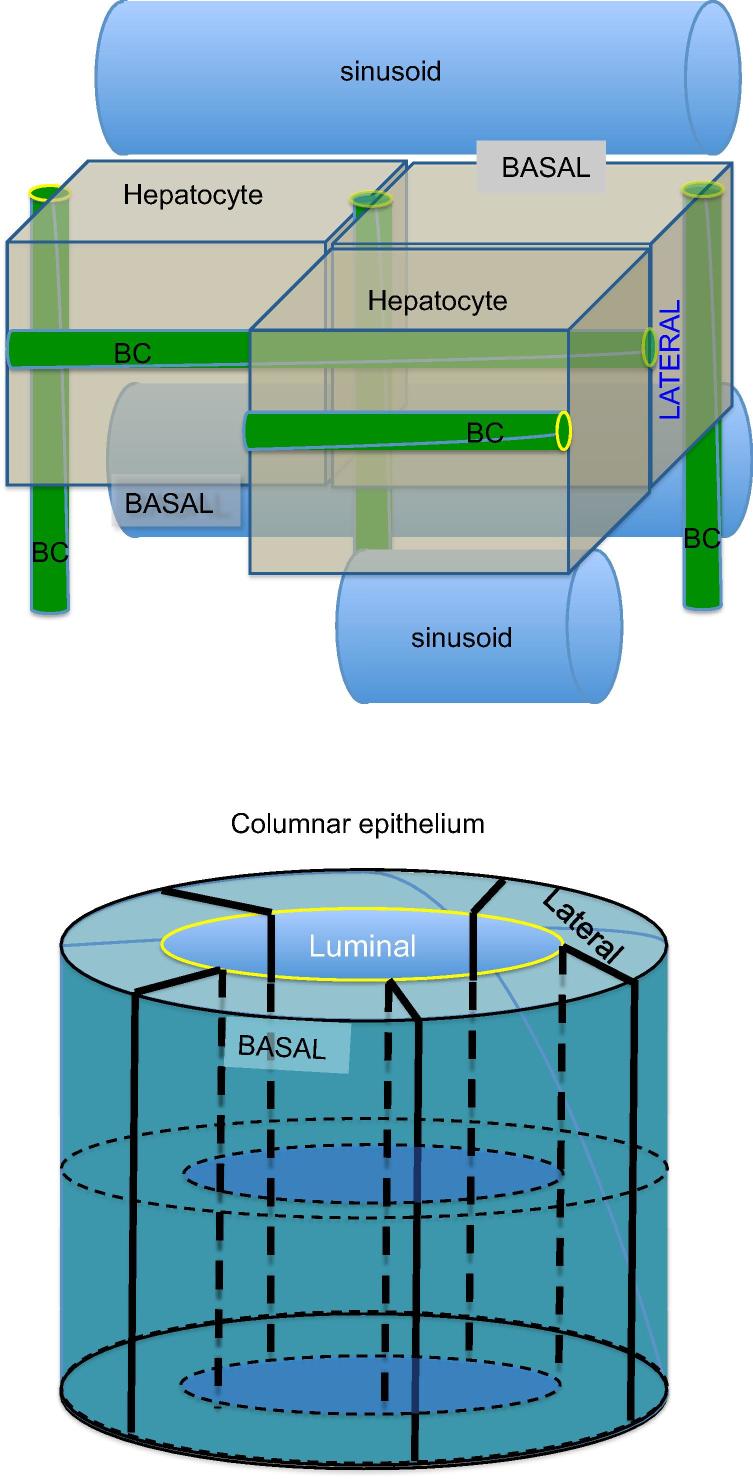
**Comparison of hepatocyte and columnar epithelial phenotypes.** (A) Adjacent hepatocytes form bile canaliculi (green) at their cell-cell contacting domains (blue) and are strengthened by surrounding tight junction belt (yellow). A single hepatocyte can form bile canalicular lumina with three neighbours (BC). Hepatocytes can also have two basal domains that face the adjacent sinusoids. (B) Columnar epithelia feature a central lumen formed by the apical domains of individual cells, which are perpendicular to their cell-cell contacting domains (black) and separated from the latter by tight junctions (yellow). The basal domains are in contact with a basal lamina (adapted with permission from Müsch A. Exp Cell Res, 2014) [153].

A defining feature of metazoans is the existence of polarized layers of epithelium which give rise to the three dimensional shapes of body parts and types. The formation and maintenance of a polarized epithelium is complex and requires specific cell adhesion molecules, cytoskeletal factors and intracellular trafficking components [1]. These give rise to apical and basolateral plasma membrane domains which separate interior from external environments and permit directional absorption and secretion of proteins and other solutes. Most epithelial cells, such as intestinal and renal tubular cells, are polarized in the plane of the tissue [2]. In contrast, hepatocytes have a unique polarization arrangement in which each of two adjacent cells contributes an apical plasma membrane that form one or more capillary-like structures, the bile canaliculus (BC), which is the smallest branch of the bile ductal system (Fig. 1) [3]. The BC is functionally sealed by tight junctions (TJs) and, with its microvilli, constitutes ∼13% of total hepatocyte plasma membrane [4]. Defects in hepatocyte polarization leads to major pathophysiological consequences.

**Figure:**
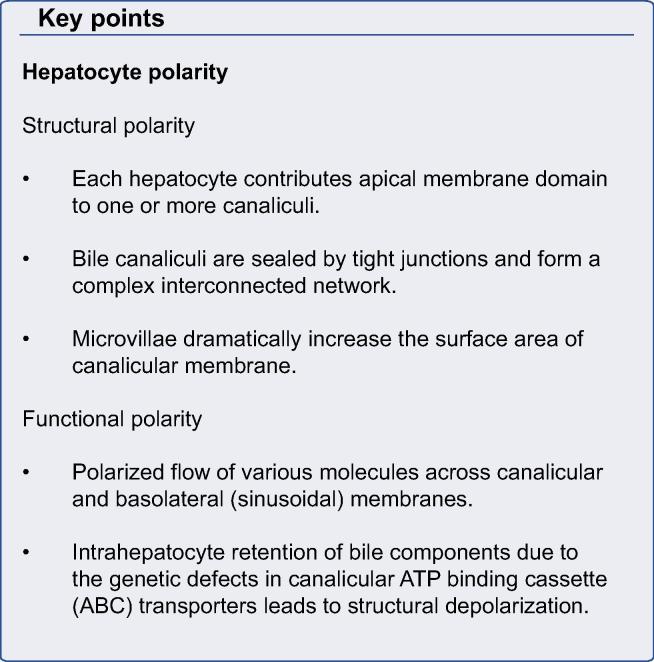

**Figure:**
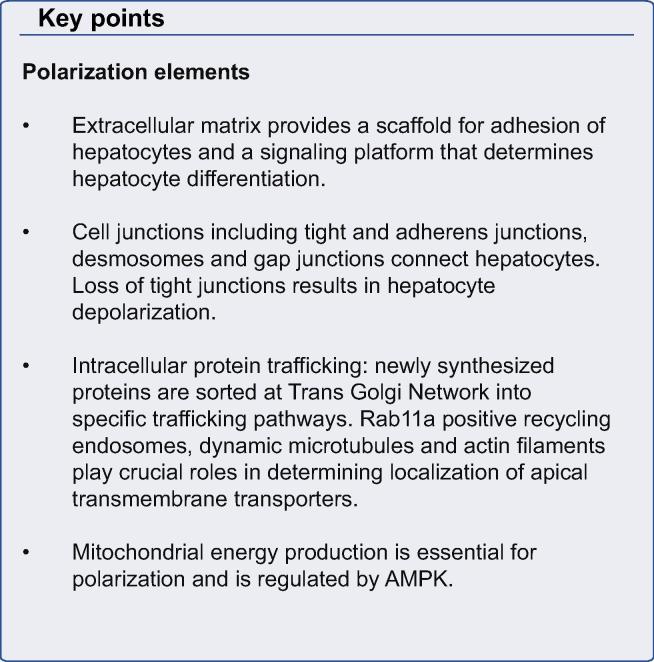

**Figure:**
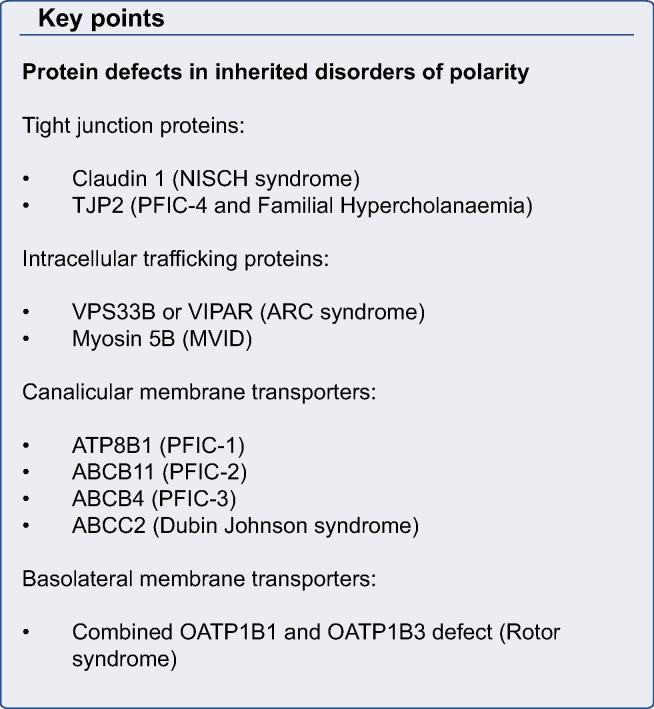

## Basic understanding of hepatocyte polarity

Hepatocyte polarity mechanisms may be divided into structural and functional components. Structural polarity includes morphologic integrity of TJs and apical plasma membranes with their microvilli, and BC network formation. In cultured hepatocytes and cell lines, reversion to planar polarity phenotype and loss of BC has been demonstrated as a result of deletion or inhibition of individual components of the complex polarization machinery [5–7]. In contrast, functional polarity is predominantly defined by the action of canalicular ATP binding cassette (ABC) transporters. The scope is expanding to include acquired diseases although pathologists rarely comment on hepatocyte polarization because BC cannot be visualized by hematoxylin and eosin staining and require imaging of canalicular proteins such as 5’nucleotidase, ABCB1, ABCB11 or others [8]. In the presence of functional polarity defects canalicular morphology is initially retained but eventually can be damaged as a consequence of intracellular retention of biliary components, particularly bile acids [9].

**Fig. 2:**
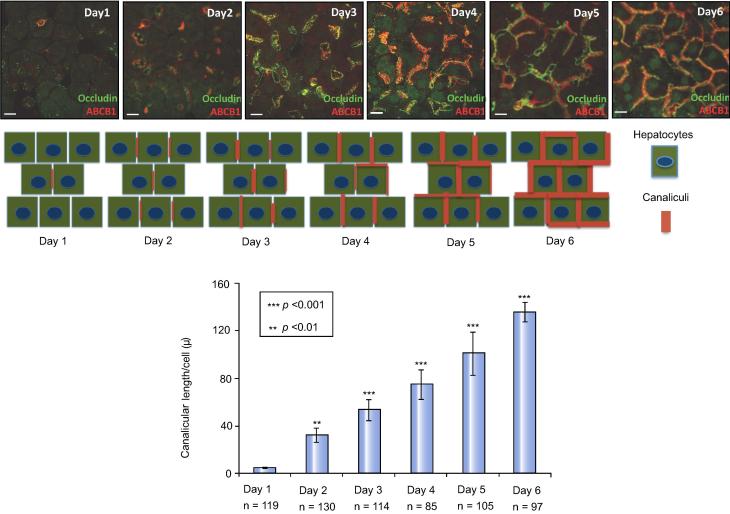
**Progressive canalicular network formation in sandwich cultures of rat primary hepatocytes.** Immunofluorescence of the tight junction marker occludin (green) and the apical marker ABCB1 (red). Diagram of canalicular network formation. Mean canalicular length (±SD) from three individual experiments (n, cell number) (adapted from Fu *et al.* J Cell Sci, 2010) [6].

Although most basic studies of components and mechanisms of polarity were identified in polarized cell lines, such as MDCK, WIF-B and HepG2; corroborating investigations in mammalian hepatocytes generally support similar mechanisms. Cell lines and primary hepatocyte cultures have significant limitations for polarization studies; however, collagen sandwich cultures of mammalian hepatocytes have proven useful because they are non-dividing, do not undergo autophagy, have stable gene expression for about two weeks and, most importantly, sequentially form a canalicular network similar to that seen *in vivo* (Fig. 2) [6,10].

### When and how does polarity become manifest?

**Fig. 3:**
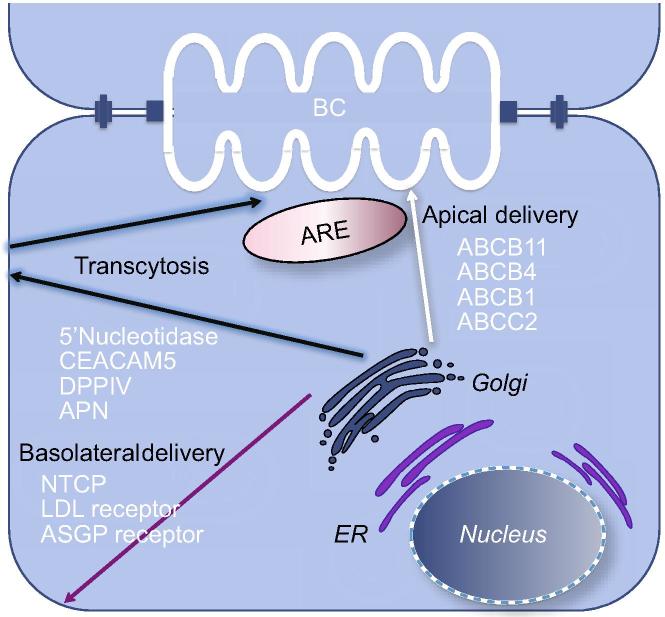
**Intracellular pathways to canalicular and basolateral plasma membranes.** Basolateral membrane proteins, including the LDL receptor, ASGP and other receptors, and NTCP, the bile acid transporter, traffic directly to the basolateral domain from which they are endocytosed and returned to the plasma membrane. The role of specific endosomal and subapical compartments in transcytosis is uncertain. Canalicular monotopic GPI-terminated proteins, mainly ectoenzymes (5NT, Aminopeptidase, CECAM105 etc) traffic from the TGN to the basolateral domain from which they undergo transcytosis through the recycling endosome (ARE) pool to the canalicular domain. In contrast, canalicular polytopic transporters ATP binding cassette proteins, such as ABCB1, ABCB11, ABCC2, and ABCB4, traffic from the TGN to the canalicular membrane either directly or via the large apical recycling endosomal (ARE) compartment from which they endogenously cycle to and from the canalicular membrane, or delivered into the degradation pathway to lysosomes. Segregation of apical and basolateral cargo proteins is thought to occur at the TGN although additional intracellular sorting sites have been proposed.

**Fig. 4:**
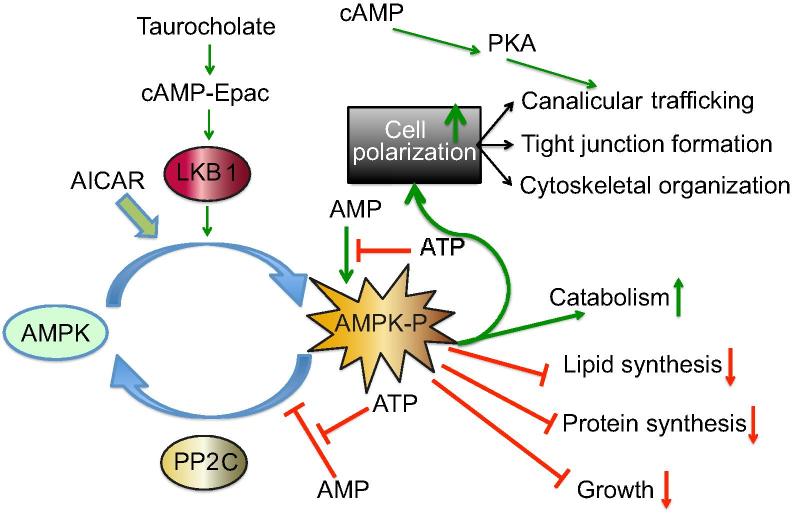
**Signaling pathways in hepatocyte polarity.** The relation between LKB1, AMPK and hepatocellular polarization is schematized based on experimental observations in sandwich cultured mouse hepatocytes (adapted from Homolya *et al.*, 2014) [22]. AMPK activation inhibits processes which utilize ATP with the exception of polarization machinery. In addition, protein catabolism is enhanced. How LKB1 participates in polarization and apical trafficking of ABCB11 and other ABC transporters is not known; however, the process is associated with AMPK activation and canalicular network formation. Taurocholate stimulates microtubular-dependent trafficking by activating the cAMP-Epac pathway, whereas, in *Lkb1*^−/−^ mice, the stimulating effect of taurocholate and Epac is prevented; however, cAMP activation restores intracellular trafficking by a PKA-dependent mechanism which is independent of AMPK. PP2C-protein phosphatase 2C removes phosphate from phosphor-AMPK. AICAR activates AMPK in a manner similar to cAMP.

Embryologically, hepatoblasts are non-polarized and give rise to hepatocytes on stimulation by Oncostatin M (OSM) and TNF-alpha, and cholangiocytes, which are signaled by NOTCH and TGF-beta [11–13]. In mice, hepatocytes begin polarization on fetal day 14; however, mature BC do not appear until fetal day 21 [14,15]. Early canalicular network occurs by day 20 and rapid postnatal network formation occurs within two-three days after birth. During development, tight junctional complexes form, and apical and basolateral proteins including transporters become associated with specific plasma membrane domains (Fig. 3) [16]. These changes are associated with activation of 7-alpha-hydroxylase and synthesis of bile acids which may participate in regulating canalicular network formation similar to effects observed in hepatocyte cultures in which bile acids acting through a cAMP-Epac-MEK-AMPK pathway accelerate canalicular network formation (Fig. 4) [7].

Acquisition of polarity requires an evolutionarily conserved interconnected network of determinants that have been discovered in *Drosophila* and which specify future polarization domains in differentiating epithelial cells [17,18]. Crumbs complex (including Crumbs, Patj, and Pals1) provides apical identity and is linked to Par complex (Par3, Par6, and alpha-PKC), which promotes TJs and apical targeting after phosphorylation of Par3 and Crumbs by alpha-PKC. Scribble complex (containing Lg1, Dlg, and Scribble) defines the basolateral domain. How these systems work in hepatocytes is unknown. The complexity of subsequent processes makes it difficult to identify specific signaling events, which explicitly shape polarization. These events, for the most part, involve protein trafficking, which has been extensively studied in canine-derived MDCK cells and to a limited extent in hepatocytes [19]. In only a few cases have the signaling events involved in canalicular network formation been demonstrated. For example, bile canalicular network formation is impaired in HNF-4alpha [20] and LKB1 knockout mice [21,22], and accelerated by STAT3 [23] and OSM [24]. OSM is an IL-6-related cytokine secreted by the hematopoietic cells in the fetal liver [25] and is able to activate transcription factors STAT3 and HNF-4alpha [23,26] as well as G-protein K-Ras [27]. OSM promoted cell-cell adhesion and adherens junction formation in cultured embryonic murine hepatic cells and bile canalicular formation in fetal human hepatocytes [28,29]. It has been shown that OSM functions in protein kinase A pathway by inducing the expression of the cell cycle inhibitor p27KIP that keeps cells in G1 and may couple centrosome-associated signaling to canalicular domain formation [24,30–32].

### The key elements in polarization

Hepatocyte polarization and canalicular network formation require coordinated expression of several key evolutionarily conserved elements each of which consists of many components. These elements are extracellular matrix (ECM), adherens and tight junctions, intracellular protein trafficking machinery including recycling endosomes, cytoskeleton and energy production.

### Extracellular matrix

The ECM is a complex macromolecular structural network which forms a scaffold for adhesion and provides a signaling platform by sequestering or releasing cytokines, anchoring processing enzymes and activating hepatocyte surface integrins which trigger intracellular signaling [33,34]. Chemical and physical properties of the ECM largely determine hepatocyte differentiation. Unlike other epithelia, hepatocytes are not attached to a tough basal lamina. Instead they are surrounded by a low density ECM, that contains hepatocyte secreted components. Mature hepatocytes are embedded in the ECM that lacks laminin which, however, is present during hepatocyte differentiation in liver development and regeneration, suggesting a crucial role for laminin during polarization [35]. The absence of a basal lamina allows the exchange of macromolecules between the sinusoid and Disse space through endothelial cell fenestra which exclude cells. In cirrhosis, excess deposition of ECM into the space of Disse results in distortion of liver architecture and abnormal hepatocyte function [36]. Collagen sandwich cultures demonstrate *in vitro* that the trapping of hepatocyte secreted ECM proteins, such as collagen IV, laminin and fibronectin, and possibly growth factors on both non-opposing surfaces is required for the development of hepatocyte polarity phenotype [6,37].

Experiments in MDCK and WIF-B cells demonstrated that Par1b, a serine/threonine kinase activated by LKB1 (see below), is a crucial determinant of hepatocyte-like polarity phenotype [38]. MDCK cells with overexpressed Par1b demonstrated hepatocyte-like polarity with interrupted staining of collagen IV and laminin at the basal surface and appearance of collagen IV and laminin at the apical surface [39]. Interestingly, seeding Par1b overexpressing MDCK cells on high collagen IV concentration ECM reverted the phenotype back to columnar [38]. In parallel, reduced expression of Par1b in WIF-B cells disrupted hepatocyte-like polarity. Studies in HepG2 cells proposed RhoA GTPase as the effector of ECM signaling that determines the position of the lumen [40,41]. Work in MDCK cells and hepatocyte cell lines established that integrins, cadherins and junctional adhesion molecule A (JAMA) act as transducers that transfer the signal from the ECM to RhoA GEFs and GAPs in order to induce the cytoskeletal changes required to establish a biliary lumen [34,42–44].

### Cell junctions

Hepatocytes connect through intercellular tight, anchoring (adherens and desmosomes) and gap junctions [45–47]. The tight junctional complex consists of several claudins, occludins, TJP (also called ZO) proteins [48], and prevents paracellular flux of molecules. Disruption of the TJs in transgenic mice [49], collagen sandwich cultured hepatocytes and tissue sections of various hepatobiliary diseases results in depolarization and eventual hepatocellular injury. In non-polarized hepatocytes and other epithelial cells, TJ proteins and some apical transporters relocate to intracellular sites including the microtubular organizing center where they colocalize with Rab11a and Myosin 5b [5]. Identification of pathogenic mutations causing disruption of TJP2 and Claudin1 in patients with severe liver disease confirms the importance of TJs in maintaining hepatocyte structure and function [50,51].

### Intracellular protein trafficking

**Fig. 5:**
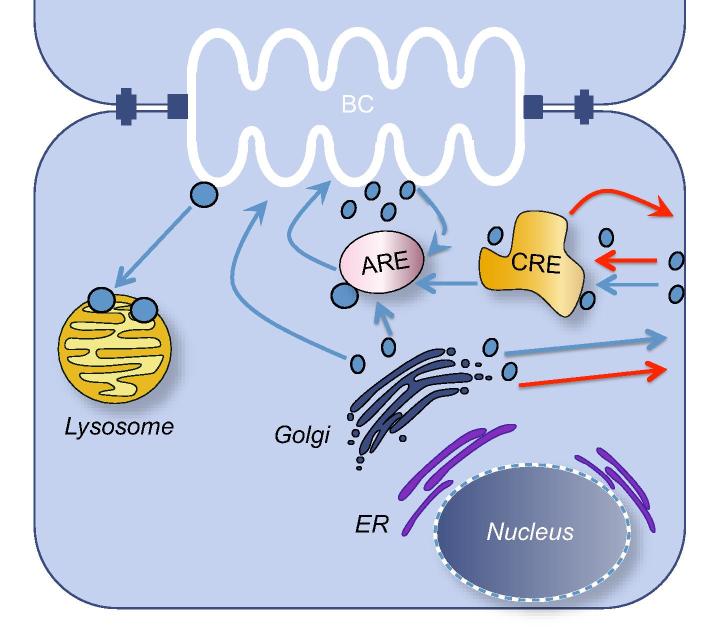
**Suggested model for intracellular trafficking pathways of plasma membrane proteins in hepatocytes.** Trafficking of polytopic apical proteins from the apical recycling endosome (ARE) compartment to the canalicular membrane is enhanced by taurocholate and cAMP, requires PI-3K, Rab11a, and Fip1 and 2 adaptor proteins, Myosin 5b and energy in the form of ATP. Endocytosis of the apical membrane proteins is clathrin-mediated and requires HAX-1, Myosin light chain kinase MLCK and most likely many other unidentified components. All intracellular trafficking requires an intact dynamic microtubular system, subsequent transfer of cargo-containing endosomes to the pericanalicular actin system, and binding to Syntaxin 3 and possibly other SNARE proteins which facilitate endosome fusion with the apical membrane. Apical membrane targeting pathways in blue. Basolateral membrane targeting pathways in red. Bile canaliculus (BC), common recycling endosome (CRE), endoplasmic reticulum (ER).

Apical protein trafficking in hepatocytes involves at least two distinct pathways (Fig. 3). Hepatocytes target polytopic membrane proteins, such as ATP transporters, from the Trans Golgi Network (TGN) either directly to the bile canalicular domain or to the Rab11a recycling endosome (RE) pool from which they cycle to the canalicular membrane [52,53]. In contrast, hepatocytes target single membrane spanning and GPI anchored bile canalicular membrane proteins to the basolateral plasma membrane from which they transcytose in endosomes through the cell to the canalicular membrane [16,53]. A specific pathway that targets copper (Cu) transporter ATP7B to the canalicular membrane via lysosome exocytosis has recently been reported [54]. It was found that in response to increasing intracellular concentrations of Cu, ATP7B is trafficked to a subset of lysosomes where Cu gets stored until the threshold concentration is reached. Once the threshold level is reached ATP7B is delivered to the canalicular portion of PM and Cu gets released. How cargo for the transport pathways is sorted at the TGN or elsewhere, and whether these pathways intersect in recycling or other endosomes has not been fully established in hepatocytes (Figs. 3 and 5). Several studies in HepG2 and WIF-B cell lines identified candidate lipid and protein determinants that contribute to sorting of canalicular proteins into distinct pathways [55–57]. The presence of different mechanisms may relate to the extensive secretion of proteins from hepatocytes into the circulation in contrast to the selective secretion of bile acids, metabolites, etc. into the bile [1]. Precise regulation of membrane sorting and of the endosomal recycling system is required to sustain hepatocyte polarity and specificity of function in the various plasma membrane domains. Approximately half of the proteins of a typical plasma membrane are endocytosed per hour, whilst only ∼8% of the hepatocyte basolateral plasma membrane is internalized per hour [58]. The difference in trafficking of different types of canalicular proteins and the importance of the Rab5 dependent endosomal pool to trafficking of GPI anchored proteins, such as DPPIV and proteins trafficked via RE such as ABCB11, was demonstrated in an *in vivo* knockdown of *Rab5* homologues. In mice with *Rab5* knockdown DPPIV remained at the basolateral membrane, whilst ABCB11 relocated to REs [59]. In contrast, the localization of ABCC2 was unaffected suggesting an independent trafficking mechanism for this protein.

Largely based on studies in cell lines and schematized in Figs. 3 and 5, the TGN is the site for release and sorting of proteins destined for the apical plasma membrane. The involvement of specific endosomal pools has not been characterized in hepatocytes but has been revealed in pulse chase experiments in liver and in cell lines. RE constitutes a large reservoir for ABC transporters which is at least six times greater than the content of those proteins in the canalicular membrane [52]. The pool is mobilized by bile acids which circulate in the enterohepatic circuit, and by postprandially secreted peptide hormones which increase cAMP production in hepatocytes to cope with the increased demand for bile acid secretion. Taurocholate and cAMP activate distinct signaling pathways to mobilize ABC transporters to the canalicular domain [60]. Regulation of these two responses differs. Increase in cAMP concentration results in PKA-mediated stimulation of PI3K but not taurocholate stimulated incorporation of ABCB11 into the canalicular membrane (Fig. 4) [61].

**Fig. 6:**
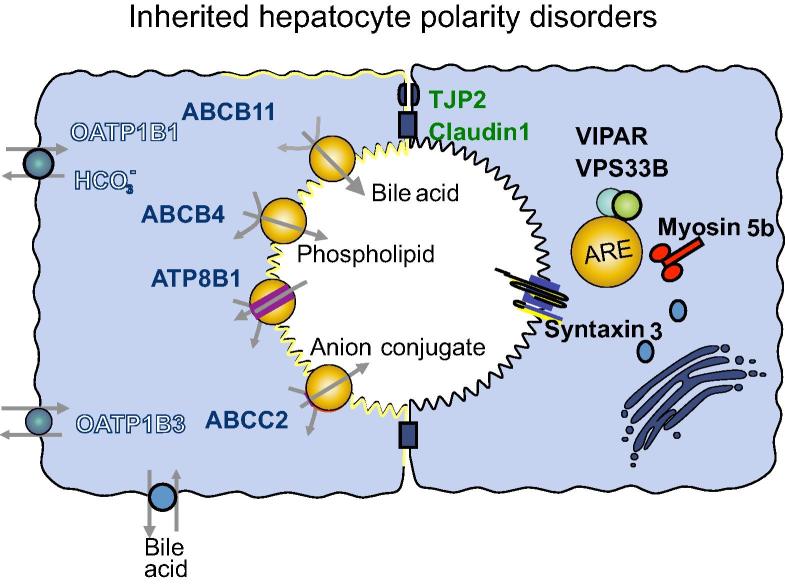
**Protein defects in hepatocyte polarity disorders.** Protein trafficking machinery proteins in yellow. VIPAR and VPS33B are associated with apical recycling endosome protein Rab11a (ARE). Unconventional motor Myosin 5b is associated with ARE and assists with trafficking along dynamic microtubules. Syntaxin 3 is a SNARE protein that acts as a docking site at the canalicular membrane. Tight junctional proteins TJP2 and Claudin1 (green) associated with a range of cholestasis syndromes. Apical membrane transporters (red) associated with inherited liver disorders include ABCB11, ABCB4, ATP8B1, ABCC2. Combined deficiency of basolateral organic anion transporters OATP1B1 and OATP1B3 (white) causes Rotor syndrome.

The RE component of the secretory pathway plays a critical role in apical as well as basolateral localization of various proteins [62]. This cargo-bearing structure contains a complex including one or more Rab protein GTPases and an actin-associated molecular motor, Myosin 5b, as well as adaptor proteins Rab11a, Fip1 and Fip2 as shown by the work in the MDCK cells [63]. Inhibition of Rab11a or Myosin 5b prevented polarization in WIF-B cells and primary hepatocytes and, when introduced into polarized cells, prompted depolarization and internalization of apical proteins [5]. These observations indicated that the RE is a major determinant of polarization not due to its ABC transporter cargo. Some of the endosome components which provide cues for apical membrane polarization have been previously characterized [64–68]. Mutations in *MYO5B* encoding Myosin 5b, which acts as a molecular motor not only for Rab11a, but also Rab11b, Rab25, and Rab8, cause MicroVillus Inclusion Disease (MVID) in which malabsorption results from the absence of the intestinal brush border [69]. Recent studies reveal that many patients with *MYO5B* mutations also manifest cholestasis and progressive liver disease [70]. Mouse Rab8 conditional knockouts mimic MVID; furthermore, patients with MVID without Myosin 5b defects were found to have mutations affecting Syntaxin 3, an apical membrane SNARE (family of membrane proteins that ensure fusion between opposing membranes), suggesting that Myosin 5b, Rab8, and Syntaxin 3 may be involved in the same trafficking pathway [71,72]. As the liver disease in mice with Rab8 deficiency or MVID patients with Syntaxin 3 defects has not been described thus far, it is possible that Rab8 and Syntaxin 3 role in this pathway is not as important in hepatocytes as it is in the intestine. Discovery of loss of function mutations in genes encoding RE-associated proteins such as Myosin 5b in MVID, VPS33B, and VIPAR in Arthrogryposis, Renal dysfunction and cholestasis syndrome (ARC) [73] supports the importance of the RE in establishment and maintenance of hepatocyte polarity (Fig. 6).

### Cytoskeletal microfilament and microtubular systems

Proper endosomal trafficking and recycling of proteins to all plasma membrane domains requires an intact actin and microtubular cytoskeletal system [74,75]. In particular, dynamic microtubules mediate trafficking of secreted and canalicular proteins [76]. Newly synthesized ABCB11, the canalicular bile acid transporter, and other canalicular ABC transporters traffic from the TGN along microtubules [77]. However, microtubules do not attach to the canalicular membrane and their cargo endosomes are transferred to the pericanalicular actin system (Fig. 5). The complete mechanism for cargo transfer is not known; however, microtubules become associated with actin through a pericanalicular actin-binding complex containing CLIP170, IQGap, APC, Hax-1, and cortactin proteins [78]. Live cell imaging studies reveal that selective plasma membrane localization of transporter proteins is predominantly due to the localization of specific docking proteins. In polarized WIF-B cells, ABCB11 and ABCB1 were shown to traffic along microtubules throughout the cell but only attach to sites on the canalicular membrane [77]. The docking site has been proposed to be Syntaxin 3 that facilitates fusion of protein sorting vesicles with the inner leaflet of the canalicular membrane [79,80]. Radixin also participates in this process and links some cargo molecules such as ABCC2, to the pericanalicular actin system [81]. Radixin knockout mice manifest impaired ABCC2 localization to the canalicular domain which becomes progressively devoid of microvilli resulting in hepatocyte injury [82]. Assembly and disassembly of short actin filaments involved in endosomal transport are under the control of formin [83]. Work in HepG2 cell line demonstrated the requirement for INF2, CDC42 and transmembrane protein MAL2, for trafficking of canalicular membrane proteins in the transcytotic pathway.

### Energy

Hepatocyte polarization is energy-dependent but the mechanism is unclear. AMPK, a serine threonine kinase containing a catalytic alpha subunit and a regulatory beta and gamma subunits, controls energy metabolism within cells by sensing the cellular AMP to ATP ratio [84]. Activation of AMPK by phosphorylation of the alpha subunit Thr172 decreases energy consumption and increases energy production during cellular stress such as hypoxia, glucose deprivation and ischaemia, and has an important role in hepatic metabolism through effects on glucose, lipid and protein homeostasis and mitochondrial biogenesis (Fig. 4) [85]. Long-term effects involve regulation of the glycolytic and lipogenic pathways [86]. In collagen sandwich hepatocyte cultures, AMPK activation by metformin, cAMP activators, 2 deoxyglucose, AICAR or taurocholate increased canalicular network formation [6]. Phosphorylation of AMPK Thr172 is performed by LKB1, an upstream serine threonine kinase which is activated by various growth factors [87]. AMPK and LKB1 regulate polarity in *Drosophila*, polarized cell lines, neurons and hepatocytes [86]. In collagen sandwich cultured hepatocytes, the stress of isolation resulted in depolarization, ATP depletion and mitochondrial fragmentation [7,88]. Mitochondrial fusion occurred within two days associated with increased ATP synthesis from oxidative phosphorylation and canalicular network formation. Subsequent AMPK activation upregulated glucose uptake, glycolysis and a further increase in ATP. These *in vitro* studies reveal that, after stress, hepatocytes preferentially restore polarity even at low ATP levels, suggesting that polarity is a prime requirement for cellular activity [7]. Mitochondrial fission and fusion are important in polarity maintenance. In other studies, LKB1 conditional liver knockout mice reveal polarity defects, cholestasis and liver injury [21,22]. Whether LKB1 and/or AMPK enhance polarization through direct effects on mitochondrial bioenergetics and/or specific phosphorylation of downstream polarity components, such as TJs, RE or cytoskeleton, is unknown.

## Disorders of polarization

Typically, diseased liver loses its polarized structure. Polarity defects are seen in single-gene rare inherited disorders (Table 1 and Fig. 6) as well as common infections such as hepatitis C, in which damage to hepatocyte polarization can be restored by treatment [89], and multifactorial diseases like cancer, as genes involved in polarity have been implicated in its pathogenesis (Table 2) [90].

### Inherited defects in junctional proteins

Defects in several apical junction proteins have been associated with inherited human liver diseases (Fig. 6). *CLDN1* and *TJP2* encode integral TJ proteins; mutations in *CLDN1* were found in neonatal ichthyosis-sclerosing cholangitis syndrome (NISCH) and *TJP2* mutations cause familial hypercholanemia (FHC) and a newly described subtype of Progressive Familial Intrahepatic Cholestasis syndrome (PFIC-4) [50,51,91]. Patients with NISCH are born with generalized skin, hair and nail abnormalities. The liver disease is said to affect both cholangiocytes and hepatocytes and the biopsy appearance varies even in patients with the same mutation, and can demonstrate typical sclerosing cholangitis features or non-specific hepatocellular and canalicular cholestasis with normal bile ducts [92]. The variability of phenotype expression suggests that variants in other genes may influence liver disease severity. The likely mechanism for liver injury is increased paracellular permeability due to abnormal TJ formation, resulting in bile regurgitation [93].

FHC patients have elevated serum bile acid concentrations, pruritus and fat malabsorption. FHC can be caused by defects in several proteins including TJP2 and also enzymes involved in bile acid biogenesis. FHC patients have normal liver enzymes apart from increased serum alkaline phosphatase activity, and variable findings on liver biopsy that include canalicular cholestasis and minimally active chronic hepatitis. Symptoms usually respond to treatment with ursodeoxycholic acid. Some patients have a combination of homozygous missense V48A mutations in *TJP2* with a heterozygous *BAAT* (Bile acid-CoA:amino acid N-acyltransferase) M76V mutation (which in the homozygous state can also cause FHC).

Frame shift deletions and duplications, and splice site *TJP2* mutations identified in all patients with PFIC-4 so far are predicted to result in the absence of the protein product [51]. The described patients had normal gamma glutamyl transpeptidase (gamma-GT) cholestasis and progressive liver disease course, with most children requiring lifesaving early liver transplantation. Two patients with severe *TJP2* mutations were reported to have developed hepatocellular carcinoma at 24 and 26 months of age [94].

### Inherited defects in intracellular trafficking

Defects in four intracellular trafficking machinery proteins (Myosin 5b, Syntaxin 3, VPS33B, and VIPAR) cause related inherited disorders with polarity defects [69,70,72,73]. Abnormalities in Myosin 5b and recently described defects in Syntaxin 3 cause MVID which typically present in infants with intractable diarrhea and characteristic features include hypoplastic villous atrophy with intracytoplasmic inclusions of brush border microvilli detected by electron microscopy. Many patients with MVID develop cholestatic liver disease similar to that in PFIC-1 and -2 [70]. Cholestasis becomes particularly prominent when parenteral nutrition is introduced. Immunostaining of ABCB11 and Rab11a in liver biopsies from patients with Myosin 5b defects demonstrated abnormal distribution of these proteins, suggesting that mislocalization of ABCB11 due to abnormal trafficking could be responsible for the liver disease, at least in this subgroup of MVID [70]. Liver disease has not yet been described in patients with Syntaxin 3 defects. As mentioned above Myosin 5b is an actin-based molecular motor whose mechanism of action is closely linked with the RE-associated Rab family proteins, which regulate polarized epithelial protein trafficking. Syntaxin 3 is an apical membrane SNARE that may act as a hepatocyte docking site for the vesicles delivering canalicular membrane transporter proteins from the REs.

ARC is an autosomal recessive multisystem disorder caused by mutations in *VPS33B* and *VIPAS39* encoding VPS33B (vacuolar protein sorting 33 homologue B) and VIPAR (VPS33B interacting protein, apical-basolateral polarity regulator). Characteristic presentation of ARC includes neonatal cholestatic jaundice, renal tubular acidosis, arthrogryposis and severe failure to thrive. Patients have normal gamma-GT but significantly increased alkaline phosphatase activity, consistent with the canalicular damage [95]. Patients’ liver biopsies show evidence of giant cell hepatitis, bile duct hypoplasia and accumulation of intrahepatocyte lipofuscin granules. Most patients described so far carry severe nonsense, splice site or frame shift mutations resulting in the absence of protein product, and fail to survive past the first year of life. However, affected children with an attenuated ARC phenotype have been identified. A splice site c.1225+5 G>C mutation in VPS33B results in production of an abnormal protein transcript that retains some function and appears to confer the mild phenotype [96]. VPS33B and VIPAR form a stable complex and interact with Rab11a suggesting a role for the VPS33B-VIPAR complex in RE trafficking pathway. Moreover, localization of bile salt export pump ABCB11, which is trafficked to canalicular membrane via Rab11a positive RE was predominantly cytoplasmic in the liver of ARC patients [73]. Localization of other canalicular membrane proteins in ARC patients’ liver was found to be variable. For example CECAM5, which is a GPI anchored protein was predominantly localized to the basolateral membrane, while the location of MRP2 in hepatocyte canaliculi was unchanged. Furthermore structural and functional abnormalities in the apical junction complex (AJC) was found in mIMCD3 cells with VPS33B and VIPAR knockdown, although the mechanism behind this defect is not clear and is likely to be caused by transcriptional downregulation of some AJC proteins such as E-Cadherin and Caludin-1 [73].

### Functional polarity defects

Defects in hepatocyte transporter proteins are the most common collective cause of inherited forms of cholestasis. A scope of severity has been demonstrated with more severe protein defects manifesting in infants, with milder abnormalities conferring susceptibility to drug induced and pregnancy induced cholestasis.

### Inherited defects in canalicular membrane transporter proteins

Progressive Familial Intrahepatic Cholestasis types I and II (PFIC-1 and PFIC-2) are characterized by persistent cholestasis with normal gamma-GT and progressive liver damage that often requires liver transplantation in childhood. Reduced concentrations of primary BA are found in bile [97,98]. A scope of severity exists in PFIC and a range of mutations resulting in the absence or mistrafficking of the proteins has been described. The majority of patients with this phenotype have mutations in *ATP8B1* (PFIC-1) and *ABCB11* (PFIC-2) although patients with a similar phenotype and mutations in *TJP2* (PFIC-4) were described recently (see above) [51,99,100]. ABCB11, also known as bile salt export pump (BSEP) is responsible for the transport of salts of primary bile acids across the canalicular membrane [101]. Patients with *ABCB11* mutations are at increased risk of hepatobiliary malignancy [102]. ATP8B1 is a member of the type 4 subfamily of P-type ATPases and is present in the apical membrane of many epithelial cells, including hepatocytes and enterocytes. It was found to translocate aminophospholipids such as phosphatidylserine (PS), from the outer to the inner leaflet of the plasma membrane bilayer [103–105]. The extrahepatic manifestations of ATP8B1 deficiency include diarrhea, recurrent pancreatitis, sensorineural deafness, delay in growth and puberty and elevated sweat chloride concentration [106].

Patients with milder missense mutations in *ABCB11* or *ATP8B1* that may confer partial instability to the protein display a benign recurrent intrahepatic cholestasis (BRIC) phenotype, in which cholestasis can completely resolve between relapses [107].

Mutations in *ABCB4* encoding ABCB4 are associated with PFIC-3 [108]. ABCB4, also called multidrug resistance protein 3 (MDR3) is a P-glycoprotein that translocates phospholipids from the internal to the external leaflet of the canalicular membrane [109]. Unlike PFIC-1, -2, and -4, PFIC-3 patients have high serum gamma-GT values, ductular reaction and early fibrosis on liver biopsy. ABCB4 deficiency cause abnormal phosphatidylcholine secretion into bile leading to hepatocyte and cholangiocyte damage due to absent emulsification of bile acids [110]. A number of cholestatic disorders have been associated with partial ABCB4 deficiency, including neonatal hepatitis and biliary cirrhosis [111,112].

Mutations in *ABCC2* cause Dubin-Johnson syndrome. Patients with this condition have recurrent episodes of jaundice without plasma bile acid accumulation. The liver biopsy demonstrates intrahepatocyte deposits of dark pigment often without any obvious hepatobiliary injury [113]. Treatment is recommended only for severe neonatal cases. Upregulation of other transporters such as ABCC3 in Dubin-Johnson syndrome patients may explain a mild phenotype [114].

*ABCC2* encodes ABCC2 or MRP2, which is a member of the multidrug resistance protein subfamily that exports anionic glutathione and glucuronate conjugates (including bilirubin) from hepatocytes into canaliculi [115]. ABCC2 is expressed on the apical membranes of many epithelial cells including hepatocytes, proximal renal tubules, gallbladder, small intestine, bronchi and placenta [116].

### Basolateral membrane protein defects

Rotor syndrome is caused by simultaneous recessive mutations in *SLCO1B1* and *SLCO1B3* genes and phenotypically is similar to Dubin-Johnson syndrome [117]. It manifests with mild jaundice that can be detected in the neonatal period or childhood. In contrast to Dubin-Johnson syndrome there are no intrahepatocyte pigment deposits present and delayed plasma clearance of unconjugated bromsulphthalein can be found [118]. *SLCO1B1* and *SLCO1B3* encode organic anion transporting polypeptides OATP1B1 and OATP1B3, which localize to the sinusoidal membrane of hepatocytes and mediate sodium-independent cellular uptake of multiple compounds, including bilirubin glucuronide, bile acids, steroid and thyroid hormones, as well as numerous drugs [119].

## Hepatocyte polarity determinants and acquired liver diseases

### MicroRNA

MicroRNAs (miRNAs), are small 18–24 nucleotide noncoding RNAs that regulate gene expression by binding to mRNAs and interfering with translation [120,121]. Typically, miRNAs downregulate expression of their target genes by binding to the 3′ untranslated region (UTR). However miRNAs may also upregulate target gene expression when interacting in a non-3′ UTR-dependent fashion. More than 1000 mammalian miRNAs are known [122]. The miRNA-target gene interaction is complex and probably exist as part of carefully regulated transcription factor networks [123]. More than one miRNA can affect expression of the same gene and each miRNA can influence dozens of gene transcripts. The exact roles of miRNAs in the establishment and maintenance of hepatocyte polarity are not known, but evidence is emerging that miRNA influence expression of adherens and TJ proteins and cytoskeleton remodelling. miR-155 was found to be upregulated after TGF-beta induction in an epithelial cell line NMuMG which resulted in loss of polarity [124]. Moreover numerous studies identified specific alterations in miRNAs signatures in different liver diseases. ZEB1 and ZEB2, the transcriptional repressors of E-cadherin are influenced by members of the miRNA-200 family and lead to epithelial to mesenchymal transition [125,126]. Upregulation of miRNAs miR-200a and miR-200b in liver fibrosis is consistent with their influence in this disease mechanism [127]. A role in regulating the PTEN – TGF-beta axis was ascribed to a network of miRNAs (including miR-106a, miR-106b, miR-18a, miR-18b, and others) that results in EMT of hepatocytes thus suggesting potential for this miRNA network to promote neoplastic transformation of hepatocytes [128].

### Liver cancer

Cancer cells become depolarized and there is evidence for the role of disturbed polarity pathways in oncogenesis. Beta-catenin is a constituent protein of adherens junctions and is critical for establishment and maintenance of epithelial polarity. It can dissociate from the junctions and is translocated to the nucleus where it may transmit the contact inhibition signal. Mutations that lead to accumulation of intracytoplasmic and nuclear Beta-catenin were identified in more than half of patients with sporadic hepatoblastoma (HB), a malignant childhood liver tumor [129]. Such Beta-catenin translocation is likely to upregulate the Wnt signaling pathway. Furthermore mutations in *CTNNB1*, which encodes Beta-catenin are associated with increased Wnt signaling were identified in ∼ 20% of patients with hepatocellular carcinoma (HCC), including a patient with primary PFIC-2 diagnosis [130].

Inactivating mutations in APC cause familial adenomatous polyposis and approximately 10% of patients with HB have germline APC mutations [131,132]. APC is a tumor suppressor that downregulates the Wnt signaling pathway by decreasing the amount of translocated Beta-catenin. APC forms a complex with glycogen synthase kinase 3 beta (GSK3B) and AXIN1 that binds and phosphorylates cytoplasmic Beta-catenin, facilitating its ubiquitination and proteasomal degradation. Understanding signaling events underlying HCC lead to development of novel treatments. A complete resolution of tumors in an *in vivo* mouse model of HCC with Beta-catenin mutations was recently demonstrated after treatment with a “locked nucleic acid” antisense approach to inactivation of Beta-catenin driven Wnt signaling [133]. In another project, MET/ΔN90-β-catenin mutant model of HCC was employed to test the effect of *in vivo* nanoparticle mediated siRNA inhibition of integrin subunits, which slowed down progression of HCC and was proposed as a possible novel treatment for this tumor [134]. Germline mutations in *PTEN* and more recently *PIK3CA* and *AKT1* were found in patients with “Cowden” or “multiple hamartoma” syndrome, an inherited disorder that leads to multiple hamartomas including biliary hamartomas, and predisposes patients to several types of cancer. PIK3CA encodes the catalytic subunit of PI3K, which adds a phosphate to phosphatidylinositol-4,5-biphosphate (PIP2) to form phosphatidylinositol-3,4,5-triphosphate (PIP3) at the cellular membrane. PTEN dephosphorylates PIP3, which is required for recruitment of AKT1 to the cell membrane where it is phosphorylated. The PIP3 pathway is important for apical membrane formation. PI3KCA somatic mutations are also occasionally seen in HCCs and other cancers [135].

### Viral hepatitis

Hepatitis B and C both promote hepatocarcinogenesis, which is associated with E-cadherin downregulation and Beta-catenin activation. Hepatitis C virus (HCV) enters hepatocytes using the TJ proteins claudin1 and occludin as co-receptors and a tetraspanin CD81 [136–139]. Hepatitis B virus (HBV) entry into hepatocytes is dependent upon hepatocyte polarization and it is suggested that the putative viral cell receptor is located in the basolateral membrane, although its identity is not yet known [140]. HBV X protein is thought to activate the Wnt signaling pathway by binding to APC [141].

The work in hepatoma cell lines demonstrated that HCV infection upregulates VEGF which disrupts tight junction integrity promoting viral transmission [89].

HCV core protein expression was associated with disrupted apical polarity in MDCK cells, which may be a result of deactivation of PI phosphatase SHIP2 which converts PtdIns(3,4,5)P3 to PtdIns(3,4)P2 [142]. Furthermore, HCV-induced liver inflammation is associated with upregulation of Wnt signaling and increased miR-155 expression [143]. Expression of another HCV protein, HCV-NS5A, in primary hepatic precursors and immortalized hepatocyte cell lines led to epithelial to mesenchymal transition (EMT) through activation of Twist2, which regulates EMT [144].

HCV entry into via hepatocyte TJs was targeted by the novel approach to therapy. Inhibition of HCV entry using anti-CLDN1 antibody in human liver-chimeric mice successfully treated chronic HCV infection and provides a novel therapeutic approach to this devastating disease [145].

### Primary biliary cirrhosis

Genome wide association studies are helping to unravel pathways involved in the pathogenesis of common human diseases. Primary biliary cirrhosis is the most common autoimmune liver disease primarily affecting women over the age of 40. Recent studies found significant association between defects in several molecular pathways and primary biliary cirrhosis, including known polarity pathways such as mTOR, PI3K, MAPK, Hh, and Wnt signaling, and adherens junctions [146]. In cirrhosis and inflammatory hepatic diseases, depolarization of hepatocytes occurs; however its frequency and contribution to pathology have not been studied in depth. A recent study has shown that induction in oxidative stress by administration of carbon tetrachloride disrupted Par-3 – alpha-PKC complex formation resulting in disassembly of TJ and depolarization of hepatocytes. These changes led to cholestasis and cirrhosis [147]. The mobilization of fibrogenic cells has been proposed to involve conversion of polarized hepatocytes and biliary epithelial cells into mesenchymal cells with concomitant loss of epithelial polarization and acquisition of a mobile phenotype, but this is controversial [148,149].

## Conclusions

Improved understanding of hepatocyte polarization benefited from the work in cell lines, model organisms and discoveries of inherited mutations in patients with cholestatic diseases. The unique role of the canalicular membrane in bile acid secretion and the deleterious intracellular effects of their retention prompt the hypothesis that links polarity, bile acid retention, mitochondrial damage, energy metabolism and cholestasis. Hepatocyte polarization and canalicular membrane formation are likely to be dependent upon stimulation by bile acids and LKB1 phosphorylation of AMPK and Par1b, as well as activation of STAT3 and TNFα transcription factors by cytokine OSM. The unique importance of ECM composition and localization in hepatocyte differentiation has been further defined by research efforts in the liver regeneration field. Hepatocytes have specific trafficking pathways for GPI anchored and single transmembrane domain canalicular membrane proteins that utilise the transcytotic route while polytopic ABC transporters traffic from TGN, either via RE or directly to the canalicular membrane. Cholestasis of various etiologies eventually results in inhibition of the direct and transcytotic protein trafficking pathways resulting in bile acid retention within hepatocytes and damage to mitochondria, Golgi and other organelles [150–152]. Interdependence of polarization and mitochondrial damage may well underlie cholestasis associated with viruses, drugs, shock and other factors.

Identification of the molecular defects responsible for pathogenesis of rare and common diseases affecting liver polarity provides desirable targets for drug design, and although there are no known therapeutic agents that can restore hepatocyte polarity, the research into drug development has already benefited from the understanding of the involvement of polarity factors in disease. Researchers have successfully tested an antisense oligonucleotide approach to treating HCC in an *in vivo* model with Beta-catenin mutations. This treatment can potentially benefit more than 20% of patients with this tumor that is driven by Wnt signaling activation. Furthermore blocking HCV entry via claudin1 with a monoclonal antibody approach eliminated chronic HCV infection, which is one of the most common causes of liver cancer. Better understanding of the miRNA role in liver polarity and disease will provide new RNA based approaches to treatment. For these reasons a better understanding of hepatocyte polarity and the increasing arsenal of potential therapeutic approaches give optimism for future treatment development in liver diseases.

## Financial support

P.G. is a Wellcome Trust Senior Research Fellow in Clinical Sciences (WT095662MA); P.G. is supported by the ERC Starter Grant 337057 CLOC.

## Conflict of interest

The authors declared that they do not have anything to disclose regarding funding or conflict of interest with respect to this manuscript.

## Author’s contributions

Both PG and IMA contributed to design, writing and revising the manuscript.

## Figures and Tables

**Table 1:** **Rare disorders affecting hepatocyte polarity.**




**Table 2:** **Polarity genes associated with common liver disorders.**
